# Editorial: Advances in Breeding for Quantitative Disease Resistance

**DOI:** 10.3389/fpls.2022.890002

**Published:** 2022-04-13

**Authors:** Valerio Hoyos-Villegas, Jianjun Chen, Anna Maria Mastrangelo, Harsh Raman

**Affiliations:** ^1^Department of Plant Science, McGill University, Montreal, QC, Canada; ^2^Mid-Florida Research & Education Center, University of Florida, Apopka, FL, United States; ^3^Research Centre for Cereal and Industrial Crops, Council for Agricultural and Economics Research (CREA), Foggia, Italy; ^4^New South Wales Department of Primary Industries, Wagga Wagga Agricultural Institute, Wagga Wagga, NSW, Australia

**Keywords:** quantitative disease resistance, plant breeding, genome–wide association, quantitative trait loci (QTL) analysis, pathosystem, fine mapping, pre-breeding, genomic selection (GS)

In plant breeding and genetics, traits are frequently classified into qualitative and quantitative. A qualitative trait is generally controlled by one or a few genes, whereas a quantitative trait is controlled by several genes. The effect of each of the alleles responsible for a quantitative trait is typically small when compared to the effect of the environment, making the inference of an individual genotype difficult to establish. Genetic bases of quantitative traits are characterized by a continuous distribution of phenotypes and detected by quantitative trait loci (QTL) analysis and genome-wide association studies (GWAS). In the world of disease resistance, quantitative disease resistance (QDR) has been reported in a large number of crops, and molecular markers tightly linked to quantitative resistance loci (QRL) controlling QDR.

Quantitative disease resistance is expressed when host plants exhibit a reduced disease reaction but not complete resistance. It is widely recognized that QDR provides long term host defense toward the disease, probably due to multiple genes requiring mutation for resistance breakdown as opposed to single genes as in the case of gene-for-gene resistance. However, QDR has been a longstanding challenge in the development of cultivars with durable resistance and new techniques such as GWAS could complement QTL mapping results. Emerging genetic, metabolomics, genomics, phenomics, machine learning, and synthetic biology tools could speed-up the development of new plant cultivars having quantitative disease resistance.

A collection of reports was assembled to represent achievements in understanding and improving QDR. New technologies provide avenues for measuring QDR in plant breeding populations, and new insights on plant-pathogen interactions provide new alternatives for studying QDR. Researchers around the world have made progress toward the goal of achieving QDR, and new tools technologies and knowledge to increase food productivity and sustainability using precision breeding to boost QDR.

The objective of this Research Topic was to collate articles updating the status of breeding for QDR. The interest was to provide an updated view of the science of breeding for QDR as well as the tools that have become available in the development of QDR. We received a total of 37 submissions, of which 27 were accepted into the collection. A group of 50 authors contributed to the collection. Among the accepted submissions, the following eight topics were covered: QTL mapping (5 articles), fine mapping (1 articles), genome-wide association (8 articles), genomic selection (4 articles), marker development (2 articles), pathogen-environment-genotype (2 articles), breeding and pre-breeding (3 articles), and reviews (2 articles).

## QTL Mapping

This collection reported the use of novel genetic populations for the exploration of QTL in mapping, breeding and pre-breeding populations. Karandeni-Dewage et al. screened a doubled haploid (DH) population derived from the secondary genepool of *Brassica napus* with the aim of introgressing resistance to *Pyrenopeziza brassicae*, the authors identified four QTLs that had moderate to large allelic effects. Similarly, Yu et al. studied a DH population for mapping resistance to clubroot disease, caused by the fungal pathogen, *Plasmodiophora brassicae* and used *Brassica rapa* as a source for resistance. The authors found gene-for-gene interaction with various pathotypes and identified two QTL associated with resistance.

Several QTL analysis papers in wheat diseases were published. Wu et al. studied partial resistance to five fungal isolates representing *Fusarium solani, F. avenaceum, F. acuminatum, F. proliferatum* and *F. graminearum* in field pea. The authors used a mapping population between resistant and susceptible parents and found multiple stable QTL for resistance while screening for the various *Fusarium* isolates. Wang et al. identified and validated stable QTL conferring adult-plant resistance to stripe rust (*Puccinia striiformis f. sp. tritici*), found in the Chinese landrace ”Gaoxianguangtoumai.” In particular, QTL *QYr.GX-2AS* was found to be present only in low frequency (5.3%) among 325 Chinese landraces. Roy et al., studied QTL for resistance to spot blotch in wheat. They used two bi-parental mapping populations and found several QTL having low to moderate effects.

## Fine Mapping

A significant contribution by Zhang et al. was published. Their paper focused on fine mapping of the leaf rust resistance gene *Lr65* in the spelt wheat cultivar “Altgold.” The authors delimited *Lr65*–a 0.8 cM interval and provided one simple sequence repeat marker and a high-resolution map, further reducing the region to 60.11 Kb in size.

## Genome-Wide Association Analysis

Liu et al. used a set of 240 Chinese elite cultivars genotyped using a 90 K single nucleotide polymorphism (SNP) array with the aim of finding signals associated with Fusarium seedling blight resistance. The authors found six stable QTL accounting 4.8–7.5% of the phenotypic variation. The authors also report four Kompetitive allele specific PCR (KASP) markers to enable marker assisted selection in wheat breeding programs. Rashid et al. used a panel of 396 tropical adapted (Asian environments) maize lines genotyped with 296 k SNPs using genotyping by sequencing (GBS) approach to screen for Charcoal rot resistance (*Macrophomina phaseolina*). The authors found 19 SNPs with significant associations and developed two F_2:3_ populations to validate the signals. Two QTL co-located with two of the SNP and haplotypes detected. The authors reported that many of the signals found overlap with previously reported QTL for Gibberella stalk rot resistance, thus increasing the opportunity to develop resistance to multiple stalk rots.

Mekonnen et al. studied *Septoria tritici* blotch (*Zymoseptoria titici*) in wheat and used a set of 178 bread wheat genotypes to screen for adult plant resistance and agronomic traits for 2 years. The association panel was genotyped using GBS and this resulted in 7 k polymorphic SNPs. Significant marker-trait associations were found in 27% of the marker pairs, suggesting 33 putative QTL with 5 QTL reported as novel. The putative QTL explained 2.7–13.2% of total phenotypic variation.

Kaur et al. deployed 89 backcross introgression lines between *Triticum durum* × *Aegilops speltoides* and evaluated them for spot blotch resistance, caused by *Bipolaris sorokiniana* for four consecutive years. The authors identified five QTLs linked to spot blotch. In particular, QTL *Q.Sb.pau-5B* was validated in this study, serving as a future diagnostic marker for spot blotch resistance.

Li, Y., et al. used a set of 89 bottle gourd [*Lagenaria siceraria* (Mol.)] Standl. accessions with the aim of finding significant associationsfor resistance to *Fusarium* wilt. The study genotyped the panel with 5 k SNPs and revealed a total of 10 SNPs detected in at least two environments. Aoun et al. identified 64 marker trait associations (MTA) for leaf rust (*Puccinia triticina*), 46 MTAs for stripe rust (*Puccinia striiformis f. sp. tritici*) and 260 MTAs for stem rust (*Puccinia graminis f. sp. tritici*) resistance in an elite durum wheat association panel genotyped with a 90 k SNP array. None of the signals for stripe rust found here corresponded to existing designations of resistance genes. In contrast, two and four of the signals for leaf rust and stem rust overlapped with known resistance genes, respectively.

A significant contribution by Ruan et al. can be found in this collection. In this paper, the authors aimed at finding useful fusarium head blight (FHB, *Fusarium graminearum* Schwabe [teleomorph: *Gibberella zeae* (Schwein.) Petch]) resistance in durum wheat. The researchers used 186 diverse durum wheat lines, comprised of elite Canadian cultivars, breeding lines and experimental durum wheat lines with FHB resistance. The authors found 31 QTL across all durum wheat chromosomes and one stable QTL of large effect. Also, three haplotypes of the QTL *Fhb1* were identified. This large number of signals provides a treasure trove of resources for improving FHB resistance, including durable FHB resistance.

Using a core collection from the United States Department of Agriculture, Shi et al. detected signals and implementing genomic selection toward soybean cyst nematode (SCN, *Heterodera glycines*) resistance in common bean (*Phaseolus vulgaris* L.). The authors used 315 accessions from the core collection and screened for SCN. The core set was genotyped with Infinium BeadChips, consisting of 4 k SNPs. A total of 15 accessions were found as resistant and 11 MTA were found. Additionally, the authors applied genome-wide prediction models and reported moderate accuracies for resistance to SCN, indicating the feasibility of using this framework when improving SCN resistance.

## Genomic Selection

A significant study carried out by Merrick et al. was published. The authors conducted research to optimize GS models related to both major and minor genes for resistance to stripe rust (*Puccinia striiformis* Westend. f. sp. *tritici* Erikss.) of wheat. The authors used two types of training populations composed of 2,630 breeding lines and 475 diversity panel lines, both groups were phenotyped for 4 years. Model comparisons were also conducted using major gene markers and genome-wide markers as fixed effects. Using 50 replications and a five-fold cross-validation, the models were then compared to marker-assisted selection (MAS). The authors found that GS had higher accuracies than MAS (0.72) for disease severity. In contrast, GS and major gene models did not outperform the base GS model. Different combinations of traits, population types and years resulted in increases in accuracy as well via the inclusion of major markers in the validation sets. As well, adding fixed effects under low prediction scenarios increased GS accuracy when using significant GWAS markers. This study is a significant step in the implementation of breeding efforts for improvement of QDR.

One noteworthy contribution was provided by Larkin et al. The authors used GS for forward prediction and compared naïve GS models (no covariates) and multi-trait GS (MTGS) models by predicting F_4:7_ lines for FHB resistance traits, deoxynivalenol (DON) accumulation and other traits in soft red winter wheat. They compared predictions with phenotypic performance over 2 years of selection based on selection accuracy and response to selection. The models correctly selected up to 70.1% of elite individuals, compared to 33% with phenotypic selection. The authors also measured realized response to selection for the various traits and found GS models were at least comparable to phenotypic selection for FHB. This study provides a way forward for the implementation of GS toward breeding for QDR in wheat.

Huster et al. conducted GWAS on a diversity panel of 149 snap bean pure lines and evaluated them for *Fusarium* root rot and multiple root morphological traits. The authors found five SNPs for disease severity and two for biomass, with multiple biochemical functions indicated. Genomic estimated breeding values (GEBV) were estimated across all bean lines and their correlations estimated for the development of GS models. Although low accuracies were found based on correlations, some overlap was found among lines with high GEBV and root rot resistance.

A notable submission was provided by Mphahlele et al. in the search for quantitative resistance to *Leptocybe invasa* gall wasp and fungal stem diseases such as *Botryosphaeria dothidea* and *Teratosphaeria zuluensis*. The authors deployed the *Eucalyptus grandis* EUChip60K SNP chip, a subset of 964 trees from 93 half-sib families genotyped with 14,347 SNPs. Single-step genomic best linear unbiased predictors (ssGBLUP) were used to predict parameters in the trial. The authors found a high positive genetic correlation with gall wasp tolerance moderate expected gains for traits such as diameter growth and gall wasp. This study may set future strategies for the improvement of Eucalyptus using GS.

## Marker Development

Peach gummosis has been reportedly caused by *Botryosphaeria Fusicoccumaesculi*), *Botryosphaeria rhodina* (anamorph *Lasiodiplodia theobromae*), and *Botryosphaeria obtuse* (anamorph *Diplodiaseriata*). In their study, Li, X., et al. used a previously identified QTL from a biparental population and integrated it with a GWAS and comparative transcriptome sequencing across 195 accessions and 145 k SNPs. The authors found five SNPs linked with gummosis disease resistance and located six candidate genes in the vicinity of significant SNPs. The authors also identified two highly resistant accessions as potential sources for breeding. Cucumber vein yellowing virus (CVYV) does not exhibit single gene resistance in cucumber and is transmitted by the whitefly (*Bemisia tabaci*). Due to the lack of tightly linked molecular markers, breeding for CVYV is challenging.

A study conducted by Kahveci et al. revealed that, via the use of genomics and bulk segregant analysis, KASP markers were developed for resistance to CVYV in an F_2_ mapping population and commercial lines. The authors also conducted variant analysis to generate SNP-based markers, and this resulted in a 101 kb-fine mapped region with eight putative candidate genes. Thus, the study provided crucial information and tools necessary to breed for CVYV resistance in the future.

## Pathogen-Environment-Genotype

In a study to understand the influence of elevated temperatures on resistance against phoma stem canker (*Leptosphaeria maculans*) in oilseed rape, Noel et al. investigated effects of temperature on individuals with and without race-specific resistance (*R*) genes and quantitative resistance. The experiments involved field sites and inoculation assays under controlled conditions and found that high maximum temperatures in June increased canker severity while this impact was reduced in genotypes with quantitative resistance but no *R* genes. This study suggested that the impact of high temperature is significantly reduced when quantitative resistance is present. The authors point out that there is genetic variation available to improve disease resistance under this condition. However, sustained high temperatures reduce the efficacy of QDR—a major concern in the face of global warming/climate change.

In Ozimati et al., the authors evaluated empirical and root necrosis data to determine the effectiveness of screening for Cassava brown streak disease (CBSD) in two breeding populations differing in selection cycles. The study aimed at comparing the assessments in these screening methods when the assessment was conducted by plant breeders vs. pathologists. The study found that broad-sense and marker-based heritability estimates differed widely from assessments within the two groups, with breeders resulting in a slightly higher upper limit.

## Breeding and Pre-Breeding

Emebiri et al. reported efforts on pre-emptive breeding for Karnal bunt in wheat. Karnal bunt caused by the fungus, *Tilletia indica* Mitra [syn. *Neovossia indica* (Mitra) Mundkur], is a major threat to food security, due to its use as a non-tariff trade barrier by several wheat-importing countries. The cultivation of resistant varieties remains the most cost-effective approach to manage the disease, but in countries that are free of the disease, genetic improvement is difficult due to quarantine restrictions. Using GWAS, the authors identified six DArTseq markers linked with resistance to Karnal bunt, each marker explained up to 29.5% of phenotypic variation. Using GS, the authors reported accuracies of up to 0.56, depending on whether the GS model included known QTL or used genome-wide markers. The authors conducted further research to identify elite parents with Karnal bunt resistance, leading to the identification of one ideal genotype with suitable agronomic traits.

The study in Awata et al. aimed at using backcross populations at the BC_3_F_2_ generation to identify progeny resistant to maize lethal necrosis (MLN) developed using marker-assisted backcrossing. The research group used SNP based markers linked to six QTL for resistance through screening 2,400 BC_3_F_2_ lines using a KASP platform. The authors found 56 BC_3_F_2_ lines that had major resistance for MLN and confirmation experiments in the field resulted in 19 lines with high levels of MLN resistance. These validated KASP markers linked to the two major QTL which will serve to speed up breeding.

Ballén-Taborda et al. used wild relatives of peanut to conduct marker-assisted backcrossing of two loci controlling resistance to peanut root-knot nematode (PRKN, *Meloidogyne arenaria*) from *Arachis stenosperma*. The study performed four cycles of backcrossing while utilizing SNP analysis for foreground selection. A population of 271 BC_3_F_1_ lines was genotyped to determine introgression level across the peanut genome. The results indicate that PRKN resistance was validated in BC3F_3_ lines with seed size characteristics maintained. The study concluded that it is the introgression of both loci validated from *A. stenosperma* that confer the resistance. The work will represent a significant step in breeding for PRKN resistance into elite peanut cultivars.

## Reviews

Soares et al. reviewed current progress in the improvement of disease resistance to *Mycosphaerella fijiensis* Morelet [anamorph: *Pseudocercospora fijiensis* (Morelet) Deighton] in bananas (*Musa* spp.). With the use of pre-established exclusion and inclusion criteria, the paper is a systematic review of papers collected using six scientific journal databases analyzing 3,070 published studies, identifying 24 relevant to the *Musa-M. fijiensis* pathosystem. Relevant articles found revealed that variable response to sigatoka exists among resistant and susceptible cultivars. In the case of *M. acuminata* wild diploids, resistance genes exist, and these have served as parental for new generations of improved diploids and introgression into elite cultivars. One of the highlights of the review indicates that the sequencing of the resistance genes in the *M. acuminata* genome still require functional validation across multiple omics data layers. Previously reported resistance genes have been involved in primary disease resistance pathways, such as jasmonic acid and ethylene signaling. Gene-based markers have been reported in *Musa* and are applicable for MAS. This review is a comprehensive panorama of the immune response found in the *Musa*-*P*. *fijiensis* pathosystem and summarizes some of the avenues available for breeders to undertake efforts to develop resistant cultivars.

Manze et al. provided an overview of genetic gain yield and virus resistance in Cassava over the last eight decades in Uganda. This study used 32 Cassava varieties released between 1940 and 2019 and conducted side-by-side multilocation trials in Uganda. Although disease resistance increased at an average up to 2.3% per year, fresh root yield and harvest index genetic gains have been non-significant. The authors reported some of the progress made in Cassava breeding, as well as some of the challenges that have yet to be solved, highlighting that breeding has mainly focused on protecting cassava against diseases while agronomic performance has not received sufficient attention.

## Final Remarks

Papers published in this Research Topic highlight progress made in classical and modern breeding for QDR, a trait that used to be evaluated solely based on visual symptom rating. It is known that QDR occurs probably in all pathosystems and is caused by the presence of multiple loci distributed across the whole genome of plants. The effect of each loci varies ranging from small to large and can be affected by environmental factors and plant growth stages as reported in this Topic. Additionally, quantitative disease loci (QDL) appears to be independent from the presence of qualitative resistance; thus, it is possible that breeding for QDR may not necessarily include genes for qualitative resistance. With the use of QTL and GWAS approaches, QDR can be analyzed by bi- or multi-parental QTL mapping and/or GWAS on diversity panels to identify QRLs in host plant genome that are closely associated with QDR. Dissecting QRLs could allow to isolate genes responsible for QDR. From breeding perspective, QRLs can be used for MAS to effectively develop new cultivars with durable resistance to pathogens of interest. The implementation of new tools (e.g., genomic selection) that enable accurate selection of large numbers of marker haplotypes simultaneously is a promising avenue for the accumulation of favorable alleles contributing to QDR. However, detecting and managing Genotype x Environment interaction in breeding for QDR continues to be a challenge.

## Author Contributions

VH-V: wrote the editorial. VH-V, JC, AM, and HR: served as lead topic editor for the collection. JC, AM, and HR: assisted with writing and proofreading the editorial. All authors contributed to the article and approved the submitted version.

## Conflict of Interest

The authors declare that the research was conducted in the absence of any commercial or financial relationships that could be construed as a potential conflict of interest.

## Publisher's Note

All claims expressed in this article are solely those of the authors and do not necessarily represent those of their affiliated organizations, or those of the publisher, the editors and the reviewers. Any product that may be evaluated in this article, or claim that may be made by its manufacturer, is not guaranteed or endorsed by the publisher.

